# Japanese traditional Kampo medicine bofutsushosan improves body mass index in participants with obesity: A systematic review and meta-analysis

**DOI:** 10.1371/journal.pone.0266917

**Published:** 2022-04-13

**Authors:** Kazushi Uneda, Yuki Kawai, Takayuki Yamada, Akira Kaneko, Ryuji Saito, Lin Chen, Tomoaki Ishigami, Takao Namiki, Tadamichi Mitsuma

**Affiliations:** 1 Department of Kampo Medicine, Aizu Medical Center, Fukushima Medical University School of Medicine, Aizuwakamatsu, Japan; 2 Department of Medical Science and Cardiorenal Medicine, Yokohama City University Graduate School of Medicine, Yokohama, Japan; 3 Renal-Electrolyte Division, Department of Medicine, University of Pittsburgh, Pittsburgh, Pennsylvania, United States of America; 4 Department of Cardiology, Sir Run Run Hospital, Nanjing Medical University, Nanjing, China; 5 Department of Japanese-Oriental (Kampo) Medicine, Graduate School of Medicine, Chiba University, Chiba, Japan; Ehime University Graduate School of Medicine, JAPAN

## Abstract

**Background:**

The number of people with obesity is rapidly increasing worldwide. Since obesity is a critical risk factor for cardiovascular diseases and mortality, the management of obesity is an urgent issue. However, anti-obesity drugs are insufficient in current clinical settings. Bofutsushosan (BTS, Fang-Feng-Tong-Sheng-San in China) is a traditional Japanese Kampo formula for patients with obesity. Recent basic studies have indicated that BTS potentially improves the pathophysiology of obesity. However, it is still unknown whether BTS clinically reduces body mass index (BMI) in patients with obesity.

**Methods:**

We searched electronic databases, including the Medline, EMBASE, Cochrane Library, and Japanese/Chinese/Korean databases, on June 15, 2021. We conducted a meta-analysis of randomized controlled trials to evaluate the effects of BTS on BMI, waist circumference, glycolipid metabolism, and blood pressure in participants with obesity. The primary outcome was change in BMI.

**Results:**

We included seven studies and 679 participants (351 in the BTS group and 328 in the control group). In participants with obesity, BTS significantly reduced BMI relative to controls (mean difference, MD [95% confidence interval]: −0.52 kg/m^2^ [−0.86, −0.18], *P* = 0.003). There was no significant difference in waist circumference, glycolipid parameters, or blood pressure. Sensitivity analyses showed robust outcomes for the primary endpoint, although the heterogeneity was considerable. Moreover, no serious adverse events were observed in the BTS group.

**Conclusion:**

BTS showed a potential benefit in safely and tolerably improving BMI in participants with obesity.

## Introduction

The number of people with obesity is rapidly increasing, with more than 650 million obese adults worldwide in 2016 [1, 2]. Furthermore, the global prevalence of obesity will surpass 15% by 2025 [3]. Since obesity is a critical risk factor for diabetes, dyslipidemia, hypertension, cardiovascular diseases, and mortality, the appropriate management of obesity is an essential issue [4–8].

Accumulating evidence indicates the efficacy of interventions for obesity in improving patients’ prognoses [9, 10]. Recent guidelines for obesity recommend drug treatments under appropriate lifestyle modification [11, 12]. However, the present choice of anti-obesity agents is limited in clinical settings [13, 14]. In Japan, only mazindol, an appetite suppressant, is approved for short-term treatment of severe obesity [15]. However, mazindol is challenging to use in patients with mental disorders because of its psychiatric side effects. The U.S. Food and Drug Administration has approved other anti-obesity agents. Orlistat, a pancreatic lipase inhibitor, is a representative drug for improving obesity [16]. However, orlistat also has a relatively high prevalence of gastrointestinal side effects, which limits drug adherence [17]. Therefore, an unmet medical need is emerging for new pharmacological options that are more tolerable and less restrictive.

Bofutsushosan (BTS, Fang-Feng-Tong-Sheng-San in China), a traditional Japanese Kampo formula, has been used for obese patients in east Asia over the centuries. BTS comprises 18 crude drugs: Scutellariae Radix, Glycyrrhizae Radix, Platycodi Radix, Gypsum Fibrosum, Atractylodis Rhizoma, Rhei Rhizoma, Schizonepetae Spica, Gardeniae Fructus, Paeoniae Radix, Cnidii Rhizoma, Angelicae Radix, Menthae Herba Saposhnikoviae Radix, Ephedrae Herba, Forsythiae Fructus, Zingiberis Rhizoma, Aluminum Silicate Hydrate with Silicon Dioxide, and Natrii Sulfas [18–21]. According to the Kampo concept, BTS can improve various problems, such as constipation, in obese patients. Notably, previous basic studies indicated that BTS could improve overweight, glycolipid abnormality, and hypertension [22–24]. However, there have only been small randomized control trials that have found inconsistent outcomes in the efficacy of BTS for patients with obesity [19, 20, 25]. The present meta-analysis was performed to assess the effects of BTS on body mass index (BMI) and cardiometabolic factors in participants with obesity.

## Methods

### Search strategy for meta-analysis

The present meta-analysis was performed according to the Preferred Reporting Items for Systematic Reviews and Meta-Analyses (PRISMA) statement [26, 27] and registered with PROSPERO (CRD42021291628). We searched eight electronic databases, including Medline, EMBASE, Cochrane Library, Japanese databases (Evidence Reports on Kampo Treatment and ICHUSHI), Chinese databases (China National Knowledge and Wan Fang Database), and the Korean medical database on June 15, 2021. The keywords for the search were as follows: ("Kampo" OR "Kampo medicine" OR "Medicine, Kampo" [MeSH] OR "traditional Chinese medicine" OR "TCM" OR "Medicine, Chinese Traditional" [MeSH] OR "Korean medicine" OR "Medicine, Korean Traditional" [MeSH] OR "herbal medicine" [MeSH] OR "bofutsushosan" OR "TJ-62" OR "fangfengtongshengsan" OR "bangpungtongseongsan") AND ("obesity" [MeSH] OR "overweight" [MeSH] OR "obese" OR ("over" AND "weight") OR "weight loss" [MeSH] OR ("weight" AND "loss")) AND ("randomized controlled trial" OR "rct" OR "random" OR "trial").

### Study selection

Two independent authors (KU and YK) selected studies through the following procedure. First, KU and YK extracted studies from electronic databases and screened their titles and abstracts independently. Second, the two authors reviewed the full text of all qualified studies according to inclusion and exclusion criteria in a blinded fashion. If any discrepancies occurred between the two authors, a third author (TY) tried to resolve them.

Inclusion criteria were described as follows: (i) patients with obesity (≥18 years old); (ii) RCT comparing an intervention group treated with BTS and a control group; (iii) reported data on BMI, waist circumference, glycolipid metabolism (total cholesterol [TC], low-density lipoprotein cholesterol [LDL-C], high-density lipoprotein cholesterol [HDL-C], triglycerides [TG]), and blood pressure between groups. Exclusion criteria were shown as follows: (i) animal experiments and (ii) lack of BMI data (even after contacting the authors). We only included studies written in English, Japanese or Chinese.

### Data extraction and quality assessment

After study selection, two reviewers (KU and YK) extracted data regarding BMI, waist circumstance, glycolipid metabolism, and blood pressure in the treatment and control groups from each study. We used the Cochrane risk of bias assessment to assess the degree of bias in our meta-analysis, focusing on the following factors: random sequence generation, allocation concealment, blinding of participants and researchers, blinding of outcome assessments, selective reporting, incomplete outcome data, and other metrics [28]. A third reviewer (TY) corrected any discrepancies regarding data extraction or quality assessment.

### Endpoints of meta-analysis

The primary endpoint was the absolute change in body mass index. The secondary endpoints were absolute change in waist circumstance, glycolipid metabolism, and blood pressure.

### Statistical analysis

We conducted a meta-analysis using Review Manager software version 5.4 (The Nordic Cochrane Centre, The Cochrane Collaboration). In this study, we extracted the difference in mean change and standard deviations (SDs) for BMI, waist circumference, glycolipid metabolism, and blood pressure between BTS and control groups from each study. For studies only reporting baseline and follow-up values, we calculated the average change from baseline to the end of the follow-up period between two groups from each study. To deal with the lack of SD data, we also estimated the change in SD according to the Cochrane Handbook (Cochrane Handbook for Systematic Reviews of Interventions) using the correlation coefficient (ρ) estimated by previous studies [19, 20, 25]. In our study, the mean differences (MDs) and 95% confidence intervals (CI) were estimated for statistical analysis using a random effects model [29]. Additionally, we performed sensitivity analyses for the robustness of our findings. Heterogeneity between studies was evaluated using Cochran’s Q test and *I*-squared (*I*^2^). *P* values < 0.05 indicated statistical significance. *I*^2^ values ≥ 75% represented considerable heterogeneity among the included studies [30, 31]. Moreover, we evaluated publication bias visually based on the symmetry of funnel plots.

## Results

### Literature search and listed studies

**Fig 1:**
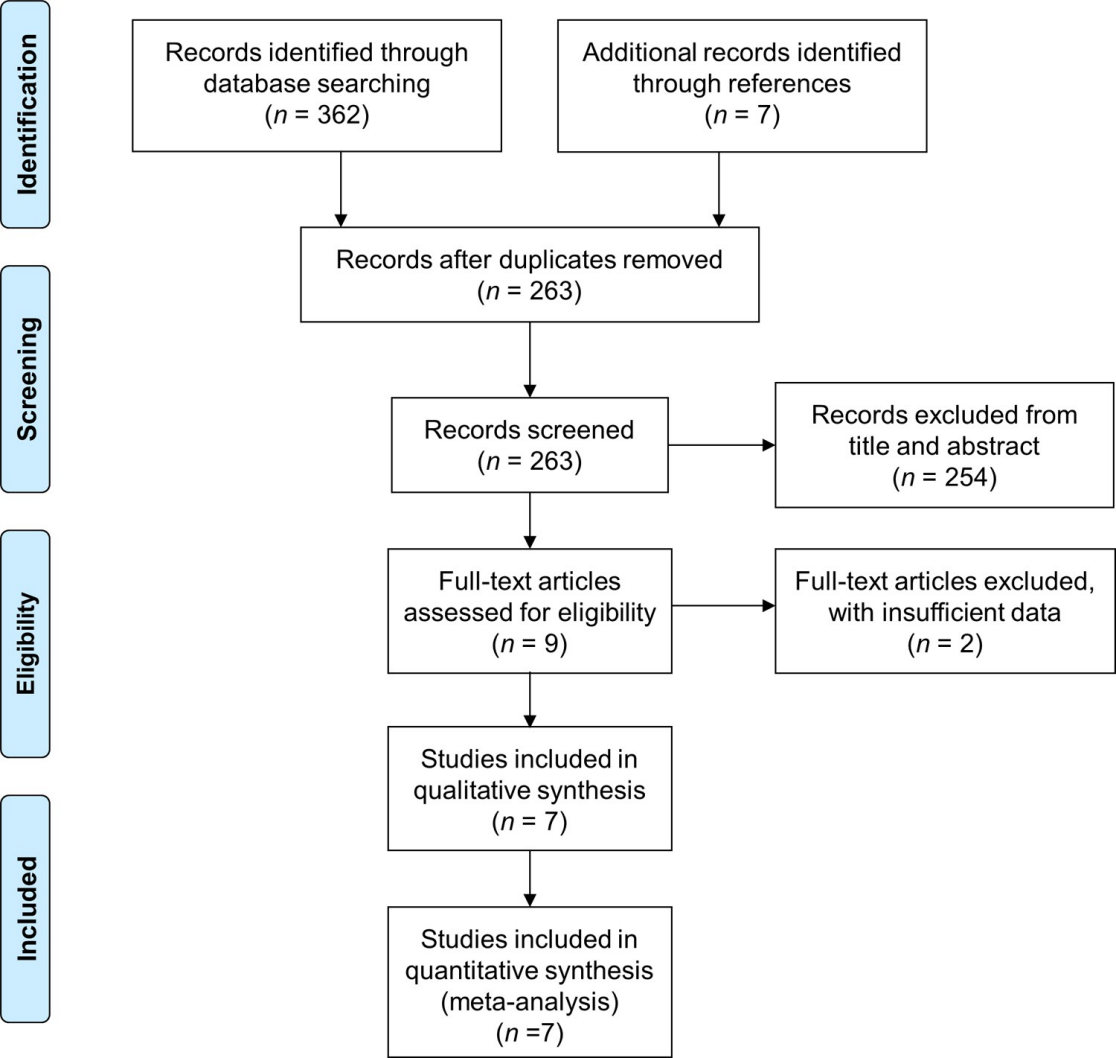
PRISMA flow chart for study selection.

Our study selection procedure is shown in Fig 1. A total of 362 studies were identified following the database search. Furthermore, seven studies were added after reviewing the reference lists of these articles. After removing duplicate studies, we screened the titles and abstract of 263 studies and excluded 254 studies. Next, we assessed the full text of the remaining nine records and removed two articles because of insufficient data. Finally, seven studies were included in our meta-analysis [19–21, 25, 32–34]. All studies were conducted as randomized controlled trials (RCTs) of BTS for participants with obesity. All studies included BMI as an outcome.

Table 1 lists the seven studies included in our analysis. Five studies were conducted in Japan, and the others were conducted in China and Korea. Three studies were double-blind placebo-controlled RCTs, whereas the others were open-label studies. One study compared BTS and another Kampo medicine, daisaikoto (dachaihutang in China). Another trial investigated the effects of adding BTS therapy to metformin treatment [21]. Three studies were performed for eight weeks, and the others were performed for 24 weeks. There was some variation in the dose of crude drugs of BTS among studies (S1 Table). Six studies defined the cut-off value of obesity as 24 or 25 kg/m^2^; one study did not mention a definition of obesity.

**Table 1:** Included studies.

Author	Year	Language	Country	Design	Groups	Definition of obesity (kg/m^2^)	Outcomes
					(I: intervention, C: control)		
Hioki et.al.	2004	English	Japan	RCT	I: BTS (dry extract) 7.5 g/day	Not mentioned	BW, BMI, WC, HC, visceral adiposity, glycolipid metabolism parameters, uric acid, resting metabolic rate, BP, subjective symptom
				24 wks.	C: Placebo (magnesium oxide 1.5 g/day)		
Namiki et al.	2007	Japanese	Japan	RCT	I: BTS (dry extract) 7.5 g/day	BMI ≥25	BW, BMI, BP, glycolipid metabolism parameters, visceral adiposity, high-sensitivity CRP
				24 wks.	C: Conventional therapy		
Wu et al.	2011	Chinese	China	RCT	I: BTS (extract) 200 mL/day	BMI ≥24	BMI, glycolipid metabolism parameters
				8 wks.	+ metformin		
					C: metformin		
Xu et al.	2012	English, Japanese, Chinese	Japan	RCT	I: BTS (dry extract) 7.5 g/day	BMI ≥25	BW, BMI, WC, W/H ratio, % body fat, glycolipid metabolism parameters, BP, high-sensitivity CRP
				8 wks.	C: Placebo (5% BTS, 95% lactose and other additives)		
Murase et al.	2013	English	Japan	RCT	I: BTS (dry extract) 7.5 g/day	BMI ≥25	BMI, home BP
				24 wks.	C: Daisaikoto 7.5 g/day		
Park et al.	2014	English	Korea	RCT	I: BTS (dry extract) 2.8 g/day	BMI ≥25	BW, BMI, WC, % body fat, body fat mass, resting metabolic rate, glycolipid metabolism parameters, BP, QOL, genome analysis.
				8 wks.	C: Placebo (corn starch)		
Azushima et al.	2015	English	Japan	RCT	I: BTS (dry extract) 7.5 g/day	BMI >25	BW, BMI, WC, glycolipid metabolism parameters, clinical and ambulatory BP, renal function, adipokine, oxidative stress
				24 wks.	C: Conventional therapy		

RCT, randomized controlled trial; wks., weeks; BTS, bofutsushosan; BW, body weight; BMI, body mass index; WC, waist circumference; HC, hip circumference; BP, blood pressure; CRP, C-reactive protein; W/H ratio, waist/hip ratio; QOL, quality of life scale.

### Study characteristics and quality assessment

**Fig 2:**
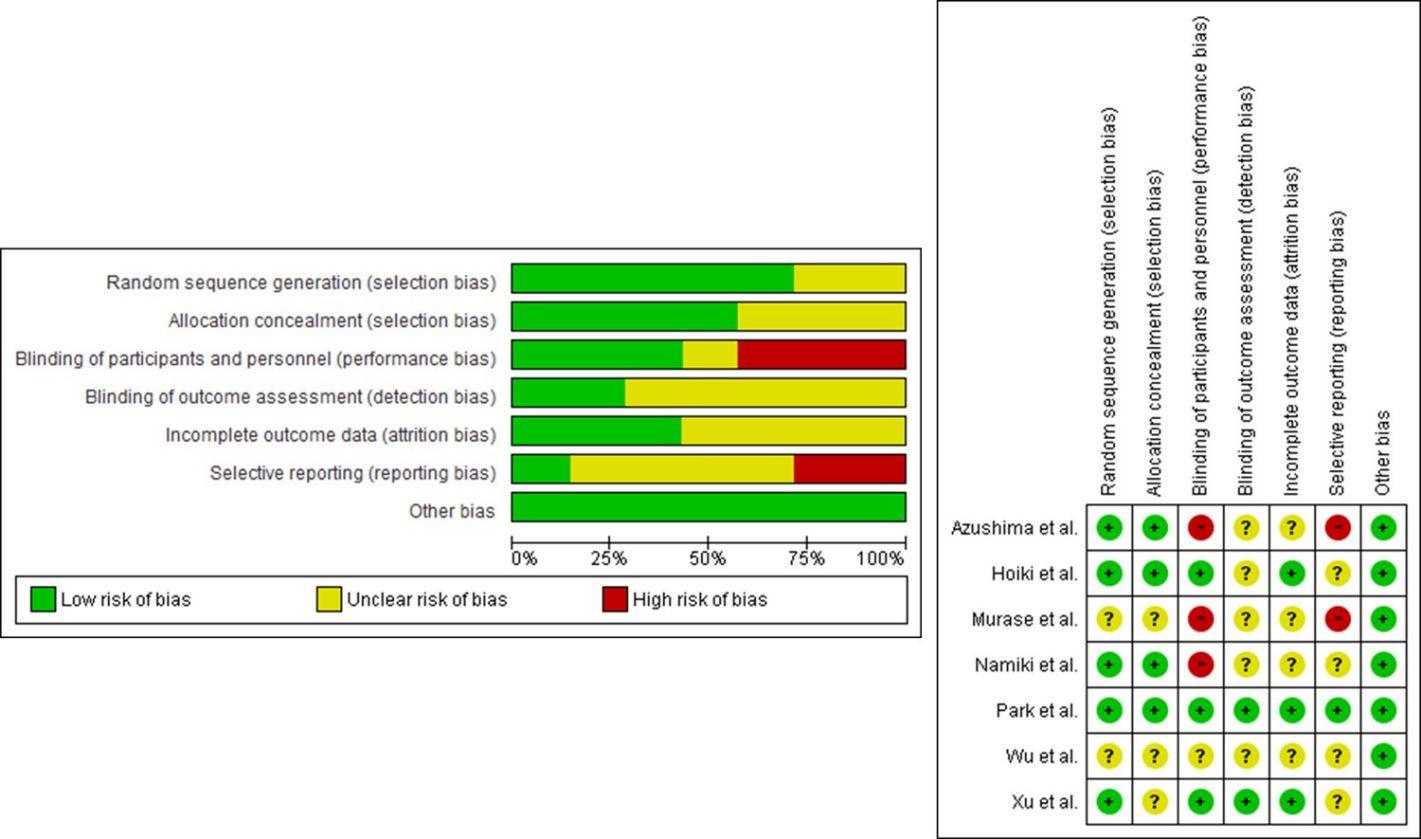
Risk of bias assessment for included studies.

The baseline characteristics of participants in the included studies are summarized in Table 2. There were 679 participants (351 in the BTS group and 328 in the control group). The mean age of participants was 53.8 years, and more than half were female. The mean BMI was 30.6 kg/m^2^. The average glycohemoglobin, LDL-C, TG, and blood pressure levels were 5.6%, 133.8 mg/dL, 166.8 mg/dL, and 136.8/84.7 mmHg, respectively. Fig 2 showed the risk of bias for the included studies. Generally, selection bias was evaluated as "low risk," although more than half of the studies had an unclear risk of bias. Performance bias was relatively high because half of the studies were planned as an open-label RCT. We could not find any publication bias in our study (S1 Fig).

**Table 2:** Baseline characteristics of participants in our study.

Study	Total participants	Age	Male	BW	BMI	WC	FBG	HbA1c	TC	LDL-C	TG	SBP	DBP
	(BTS/CTL)	(years)	(%)	(kg)	(kg/m^2^)	(cm)	(mg/dL)	(%)	(mg/dL)	(mg/dL)	(mg/dL)	(mmHg)	(mmHg)
Hioki et.al.	85 (44/41)	53.7	0	90.6	36.5	113.3	114.0	5.7	231.3	145.8	192.7	142.8	86.4
Namiki et al.	57 (25/32)	63.8	63.6	74.4	29.0	N/A	117.1	6.2	200.7	124.6	155.6	140.6	83.0
Wu et al.	72 (38/34)	46.7	50.0	N/A	26.9	N/A	169.2	N/A	235.6	N/A	201.3	N/A	N/A
Xu et al.	120 (70/50)	60.0	26.8	66.2	27.5	85.4	99.0	4.9	223.0	156.0	140.0	140.8	92.6
Murase et al.	128 (65/63)	54.8	N/A	N/A	32.8	N/A	N/A	N/A	N/A	N/A	N/A	N/A	N/A
Park et al.	111 (55/56)	40.4	14.4	75.7	29.5	98.3	98.7	N/A	199.7	124.4*	125.2	120.4	77.5
Azushima et al.	106 (54/52)	59.6	53.8	81.2	31.0	103.0	117.9	6.0	200.1	116.1	201.7	143.0	83.5
Weighted average	679 (351/328)	53.8	32.4	60.5	30.6	99.0	116.2	5.6	214.3	133.8	166.8	136.8	84.7

Data are mean values. The total number of participants in this table is defined as all participants at a randomized point in each study. BTS, bofutsushosan group; CTL, control group; BW, body weight; BMI, body mass index; WC, waist circumference; FBS, fasting blood glucose; HbA1c, hemoglobin A1c; TC, total cholesterol, LDL-C, low-density lipoprotein-cholesterol; TG, triglyceride; SBP, systolic blood pressure; DBP, diastolic blood pressure; N/A, not available.

*LDL-C data are estimated using the Friedewald formula.

### Efficacy of BTS for BMI and waist circumference

**Fig 3:**
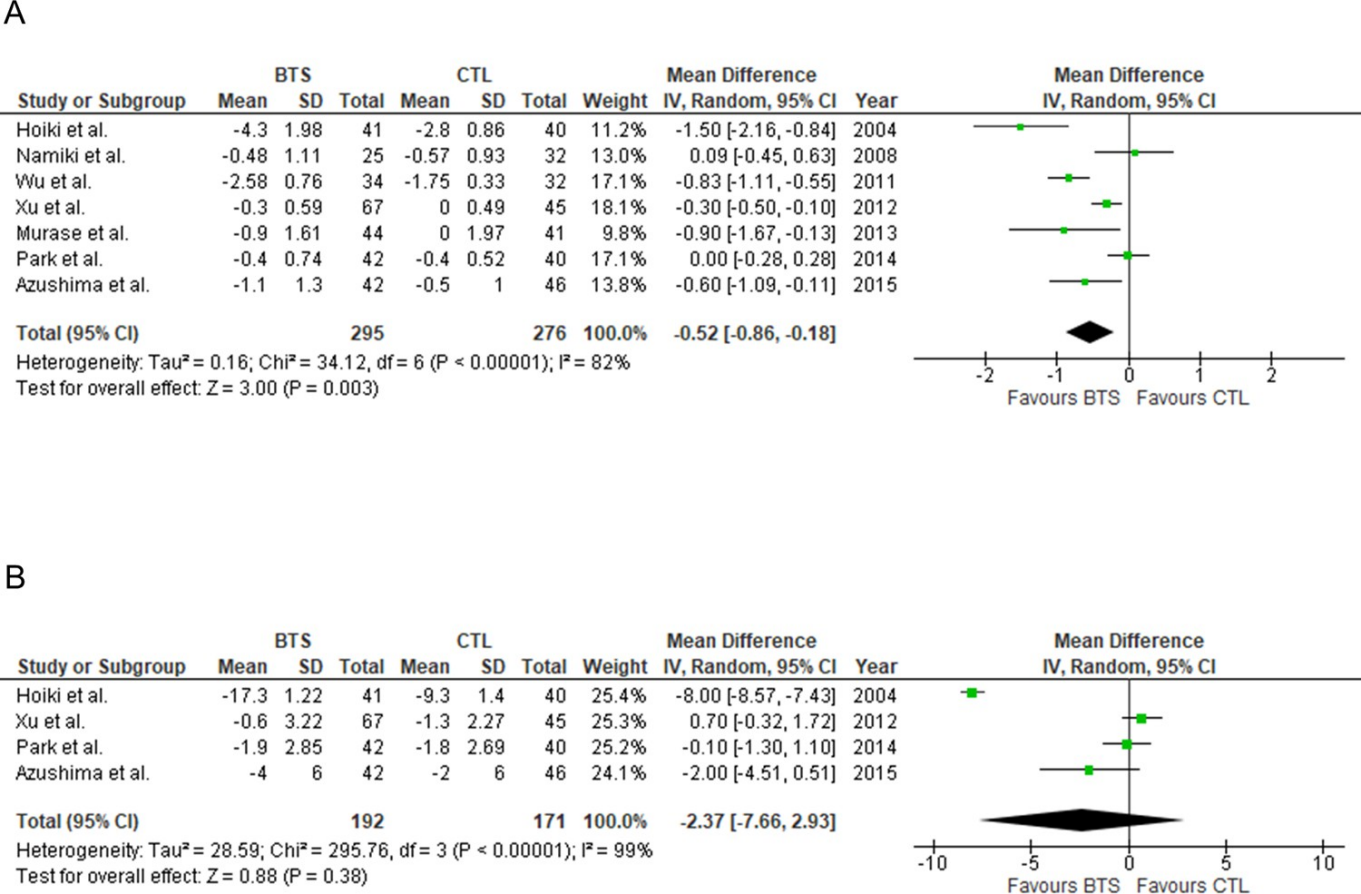
Meta-analysis of the efficacy of BTS for BMI and waist circumference reduction. (A) Body mass index. (B) Waist circumference. BTS, bofutsushosan group; CTL, control group.

We conducted a meta-analysis of all participants who completed the included studies. Fig 3 shows the effects of BTS on BMI in participants with obesity. BTS significantly reduced BMI relative to controls (MD [95% confidence interval, CI]: −0.52 kg/m^2^ [−0.86, −0.18], *P* = 0.003) (Fig 3A). Heterogeneity was considerable (*I*^2^ = 82%, *P*<0.00001). There was no statistically significant difference in waist circumference between the two groups. (MD [95% CI]: waist circumference, −2.37 cm [−7.66, 2.93], *P* = 0.38) (Fig 3B). Considerable heterogeneity was observed (*I*^2^ = 99%, *P*<0.00001).

### Influence of BTS on glycolipid metabolism and blood pressure

**Fig 4:**
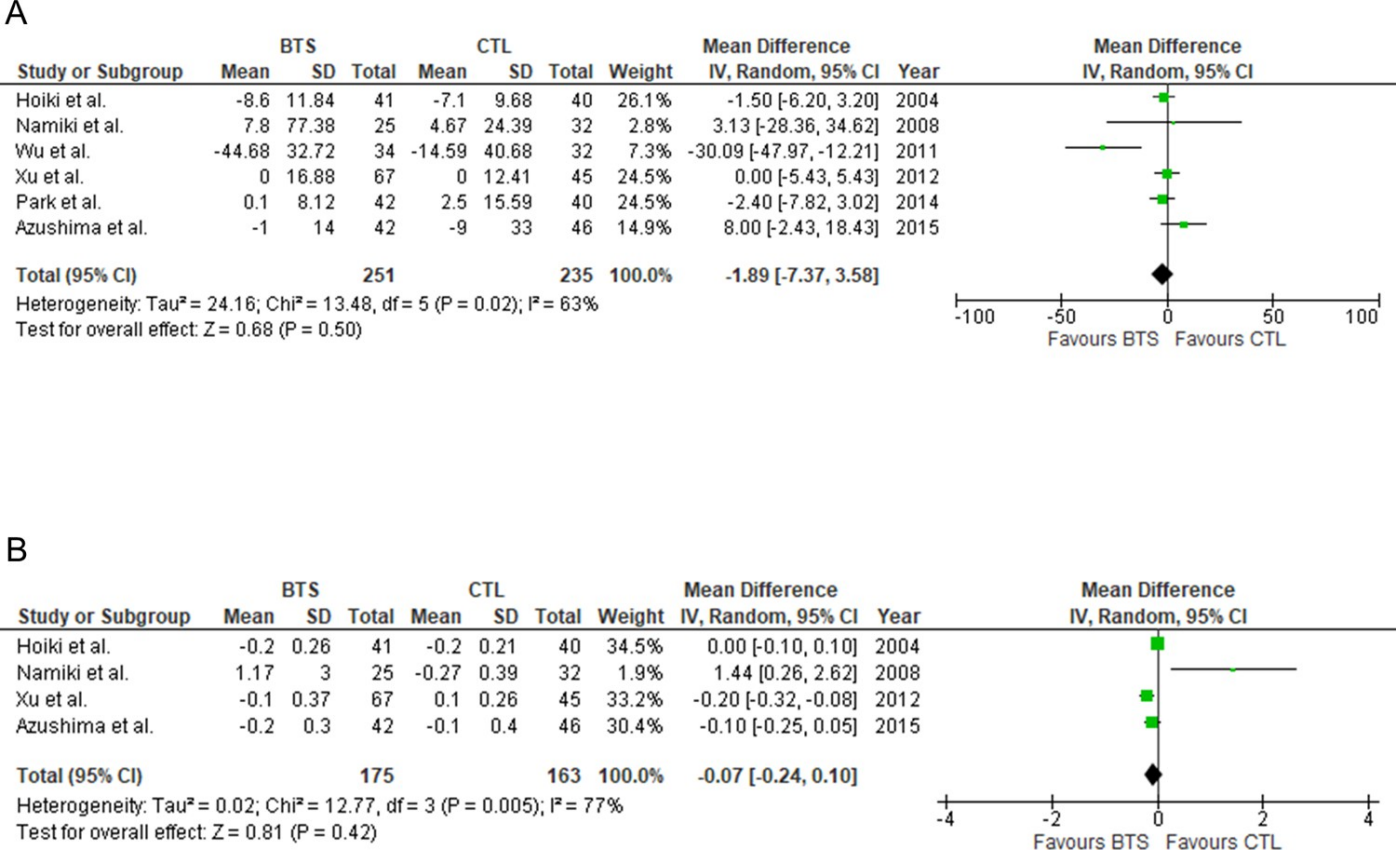
Meta-analysis of the effects of BTS on glucose metabolism parameters. (A) Free blood glucose. (B) Glycohemoglobin. BTS, bofutsushosan group; CTL, control group.

**Fig 5:**
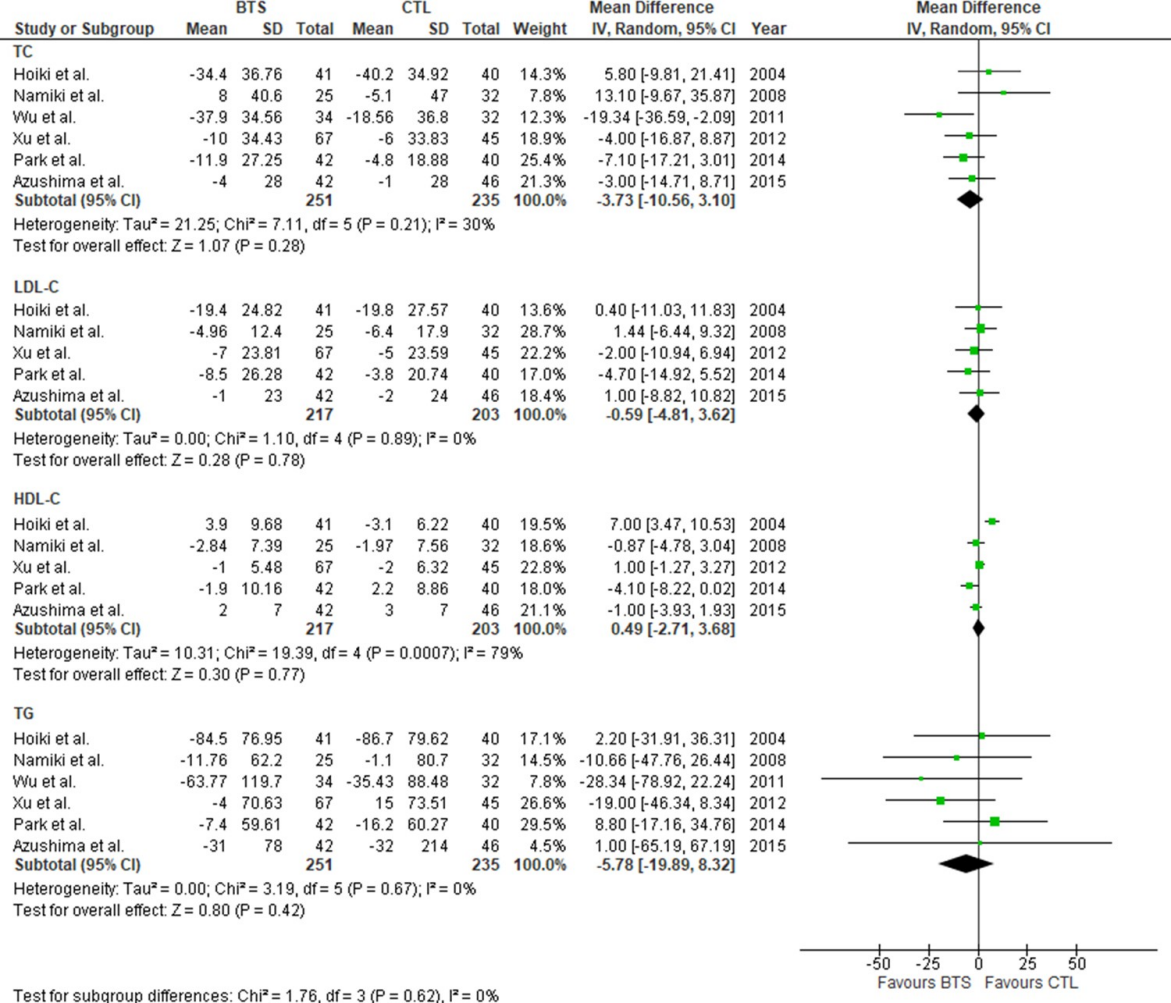
Meta-analysis of the effects of BTS on lipid metabolism parameters. (A) Total cholesterol. (B) Low-density lipoprotein cholesterol. (C) High-density lipoprotein cholesterol. (D) Triglyceride. BTS, bofutsushosan group; CTL, control group.

**Fig 6:**
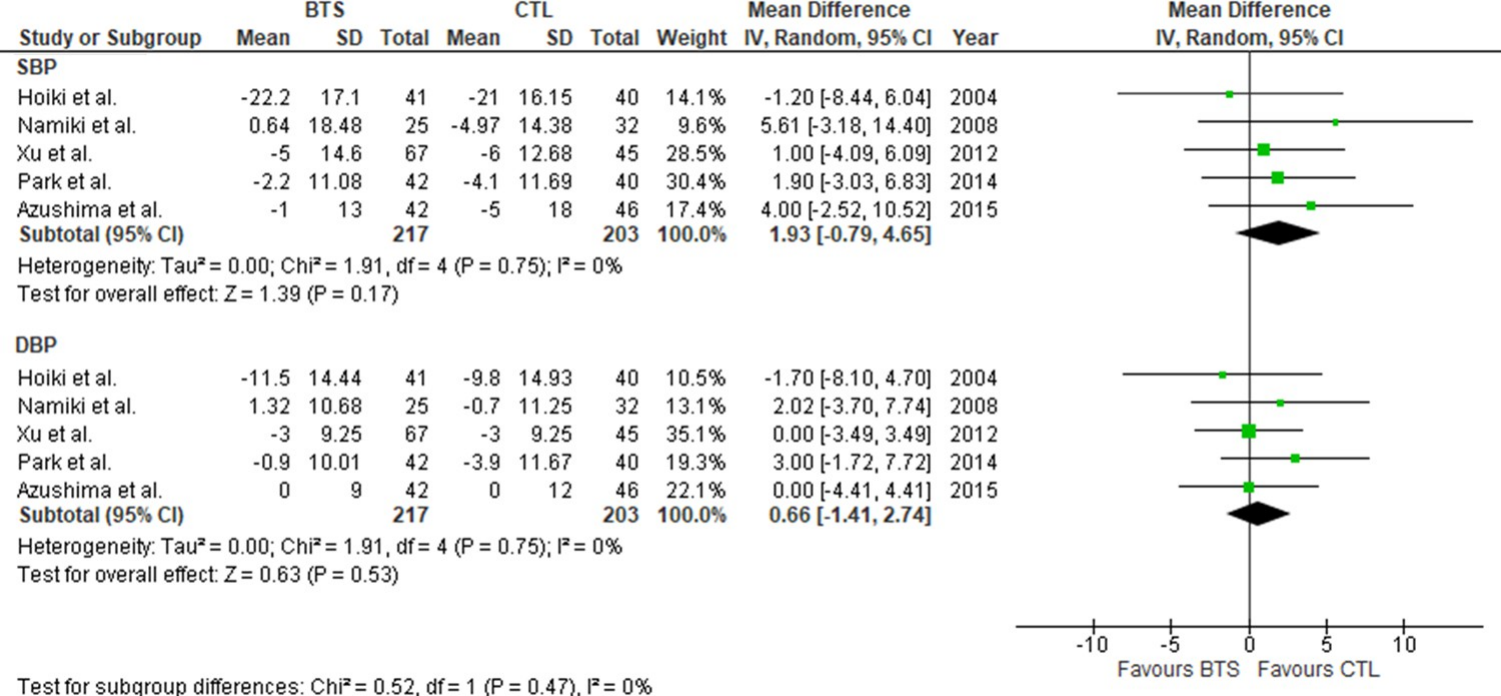
Meta-analysis of the effects of BTS on blood pressure. (A) Systolic blood pressure. (B) Diastolic blood pressure. BTS, bofutsushosan group; CTL, control group.

Next, we evaluated the effects of BTS on glycolipid metabolism in participants with obesity. BTS did not significantly reduce free blood glucose (FBG)/glycohemoglobin levels relative to controls (MD [95% CI]: free blood glucose, −1.89 mg/dL [−7.37, 3.58], *P* = 0.50; glycohemoglobin, MD [95% CI]: −0.07% [−0.24, 0.11], *P* = 0.42) (Fig 4). Considerable heterogeneity was observed in glycohemoglobin (*I*^2^ = 77%, *P* = 0.005). Similarly, there was no significant difference in lipid profile between both groups (MD [95% CI]: TC, −3.73 mg/dL [−10.56, 3.10], *P* = 0.28; LDL-C, −0.59 mg/dL [−4.81, 3.62], *P* = 0.78; HDL-C, 0.49 mg/dL [−2.71, 3.68], *P* = 0.77; TG, −5.78 mg/dL −19.89, 8.32], *P* = 0.42) (Fig 5). There was a considerable heterogeneity in HDL-C (*I*^2^ = 79%, *P* = 0.007). Additionally, Fig 6 shows the influence of BTS on blood pressure in participants with obesity. We observed no significant difference in blood pressure between the two groups (MD [95% CI]: systolic blood pressure, 1.93 mmHg [−0.79, 4.65], *P* = 0.17; diastolic blood pressure, 0.66 mmHg [−1.41, 2.74], *P* = 0.53, respectively).

### Sensitivity analyses for BMI

For robust assessment, we performed sensitivity analyses for the primary outcome (Table 3). First, we limited the analysis to studies providing a definition of obesity. BTS improved the BMI relative to that in controls (MD [95% CI]: −0.40 kg/m^2^ [−0.71, −0.08], *P* = 0.01). There was considerable heterogeneity (*I*^2^ = 78%, *P* = 0.0003). Second, we evaluated the influence of baseline BMI levels on the primary outcome. We defined the baseline weighted average BMI as a cut-off value. In participants with lower BMI, there was no significant difference in BMI improvement between BMI and control groups (MD [95% CI]: −0.29 kg/m^2^ [−0.67, 0.09], *P* = 0.14), and considerable heterogeneity was shown (*I*^2^ = 85%, *P* = 0.0002). Contrary to this, BTS significantly reduced BMI in participants with higher BMI (MD [95% CI]: −0.97 kg/m^2^ [−1.52, -0.42], *P* = 0.0006), and heterogeneity was not considerable (*I*^2^ = 57%, *P* = 0.10). Third, we compared outcomes of BTS between short-term and long-term trials. Among 8-week studies, BTS did not reach a significant difference in BMI profile between both groups (MD [95% CI]: −0.37 kg/m^2^ [−0.81, 0.06], *P* = 0.09). In contrast, among the 24-week studies, BTS reduced BMI in participants with obesity (MD [95% CI]: −0.70 kg/m^2^ [−1.35, −0.05], *P* = 0.03). Considerable heterogeneity was observed in both analyses (*I*^2^ = 89%, *P* = 0.0001; *I*^2^ = 78%, *P* = 0.003, respectively). Fourth, we considered the laxative effect of BTS on BMI reduction. Among the crude drugs of BTS, Rhei Rhizoma, a traditional laxative, could affect participants’ BMI. In our study, three trials by Hioki, Xu, and Murase adopted placebos containing laxative components. Thus, we performed a sensitivity analysis to compare the BTS and these three groups. There was a significant BMI improvement in the BTS group relative to the controls using laxative placebos (MD [95% CI]: -0.85 kg/m^2^ [−1.64, −0.06], *P* = 0.03) with considerable heterogeneity (*I*^*2*^ = 85%, *P* = 0.001). Finally, we focused on the contents of BTS. One study [21] used a BTS extract with a higher dose of crucial drugs than that in the other studies (S1 Table). We conducted a sensitivity analysis excluding that study. BTS showed significant efficacy in improving BMI in participants with obesity (MD [95% CI]: −0.45 kg/m^2^ [−0.82, −0.09], *P* = 0.01). Heterogeneity was considerable (*I*^2^ = 78%, *P* = 0.0003). These results support the efficacy of BTS in reducing BMI in participants with obesity.

**Table 3:** Sensitivity analyses for body mass index.

	Number of participants	Number of participants	MD	95% CI	Heterogeneity	
	in BTS group	in CTL group			(*I*^*2*^, *P* value)	
Only including studies defining obesity	254	236	−0.40	−0.71, −0.08	78%	0.0003
(Excluding Hioki)						
Only studies including participants with lower BMI*	168	149	-0.29	-0.67, 0.09	85%	0.0002
(Namiki, Wu, Xu, Park)						
Only studies including participants with higher BMI*	127	127	-0.97	-1.52, -0.42	57%	0.10
(Hioki, Murase, Azushima)						
Only 8-week studies	143	117	−0.37	−0.81, 0.06	89%	0.0001
(Wu, Xu, Park)						
Only 24-week studies	152	159	−0.70	−1.35, −0.05	78%	0.003
(Hioki, Namiki, Murase, and Azushima)						
Only studies using laxative components	152	126	-0.85	-1.64, -0.06	85%	0.001
(Hioki, Xu, and Murase)						
Excluding studies using high-dose BTS	261	244	−0.45	−0.82, −0.09	78%	0.0003
(Excluding Wu)						

BTS, bofutsushosan group; CTL, control group; MD, mean difference.

*A cut-off value is defined as the baseline weighted average of BMI.

### Adverse effects of BTS

The adverse effects of BTS are summarized in Table 4. Gastrointestinal symptoms were observed in 4.84% (17/351) of participants in the BTS group and 2.13% of participants (7/328) in the control group. Notably, no serious events were reported in either group.

**Table 4:** Adverse events in the included studies.

Study	Adverse events
Hioki et.al.	CTL: no serious adverse effects
	BTS: discomfort during defecation (3)
Namiki et al.	No serious adverse effects
Wu et al.	No serious adverse effects
Xu et al.	BTS: fever (1), liver dysfunction (2)
	CTL: fever (1), constipation (4)
Murase et al.	Not reported
Park et al.	BTS: gastrointestinal symptoms (12), headache (2), palpitations (1)
	CTL: gastrointestinal symptoms (3), headache (1)
Azushima et al.	BTS: gastrointestinal symptoms (2), liver dysfunction (1)

Values are number of events. BTS, bofutsushosan group; CTL, control group.

## Discussion

We present here the first meta-analysis to indicate the efficacy of BTS for improving BMI in obese participants. BTS is a traditional Kampo medicine for patients with visceral fat obesity in Japan [35]. In our study, BTS had some variation in the crude drug components. Particularly, the dose of crude drugs used in Wu’s study was higher than that in other studies [21]. However, our sensitivity analysis excluding Wu’s study showed that BMI in the BTS groups decreased significantly compared with that in the control groups. Moreover, in another sensitivity analysis, BTS improved BMI compared to placebos containing laxative ingredients. Our results demonstrate that BTS is one of the new pharmacologic options for treating obesity. Previous studies reported that BTS reduced visceral fat in experimental animals and patients [24, 36]. In our study, BTS did not lead to a significant waist circumference reduction. Further investigations are needed to clarify the efficacy of BTS in visceral fat reduction.

Previous basic studies have shown the weight loss effect of BTS through several mechanisms [24, 37–39]. Among the components of BTS, Ephedrae Herba, Glycyrrhizae Radix, Schizonepetae Spica, and Forsythiae Fructus promoted brown fat thermogenesis and white fat lipolysis in experimental animals [24, 37, 40, 41]. One clinical trial also suggested an increase in fat consumption induced by BTS treatment [36]. Moreover, BTS showed potential appetite-suppressant activity through regulating the ghrelin system [24]. Taken together, these mechanisms of BTS exerted a possible effect leading to the improvement in BMI shown in our study.

The changes in glycolipid metabolism between the BTS and control groups were not significantly different in our study. Past studies have reported that BTS improves impaired glucose tolerance and dyslipidemia in experimental animals and patients [33, 39, 42]. The mild abnormality of participants’ baseline glycolipid parameters may influence the results of our meta-analysis. Further studies on the effects of BTS on glycolipid metabolism in obese patients with diabetes and dyslipidemia are warranted. Similarly, there was no significant difference in blood pressure changes between both groups in our study. We could not find any clinical research reporting BTS lowering blood pressure, including our study. However, BTS improved hypertension, as well as body weight, in experimental obese animals [24, 39]. Further investigations are needed to explain the discrepancy in BTS’s effects on blood pressure in obesity between basic and clinical research.

Several anti-obese agents can currently be prescribed in clinical settings [11–13, 43]. However, these agents have some problems, such as side effects and administration　form. Mazindol, phentermine/topiramate, and naltrexone/bupropion suppress patients’ appetite and exert a weight loss effect [15, 44, 45]. However, these drugs are related to psychiatric adverse events, such as insomnia, anxiety, and hallucination [15, 43, 44, 46]. Orlistat is also an available anti-obesity agent. Because orlistat inhibits intestinal lipases and improves obesity, several gastrointestinal side effects, such as diarrhea or oily stool, are reported in 15–30% of participants in past trials [17]. Liraglutide, one of the representative glucagon-like peptide 1 (GLP-1) receptor agonists, exerts favorable effects on obesity and cardiovascular disease [47–49]. However, liraglutide is an injectable drug and has a possibility of mild gastrointestinal adverse events [43, 48, 49]. In contrast, BTS, an oral Kampo medicine, showed no serious adverse events in our study (Table 4). There were few reports of diarrhea, even though BTS contains Rhei Rhizoma, a traditional laxative. Therefore, these points proved the safety and good tolerability of BTS.

In our study, some limitations were observed. First, the risk of bias for some included studies was relatively high. Second, the heterogeneity related to the primary outcome was generally high. No significant heterogeneity was observed in the sensitivity analysis, including only participants with higher baseline BMI levels. Therefore, variation in participants’ baseline characteristics of BMI levels may be related to heterogeneity in our study. Third, most participants in our study were East Asians. Because previous reports have suggested the cut-off points for obesity in Asians are lower than those in other ethnicities [13], further multinational trials are warranted to investigate the anti-obesity effects of BTS.

## Conclusion

BTS safely and tolerably exerted a potential benefit to reduce BMI in participants with obesity.

## Supporting information

S1 Fig Funnel plot for risk of publication bias. MD, mean difference.
(TIF)

S1 Table Variations of crude drugs in BTS. (DOCX)
