# Supplementary figures and images for: Japanese traditional Kampo medicine bofutsushosan improves body mass index in participants with obesity: A systematic review and meta-analysis
Source: PLoS One. 2022 Apr 13;17(4):e0266917. doi: 10.1371/journal.pone.0266917 (PMC9007387; doi:10.1371/journal.pone.0266917)

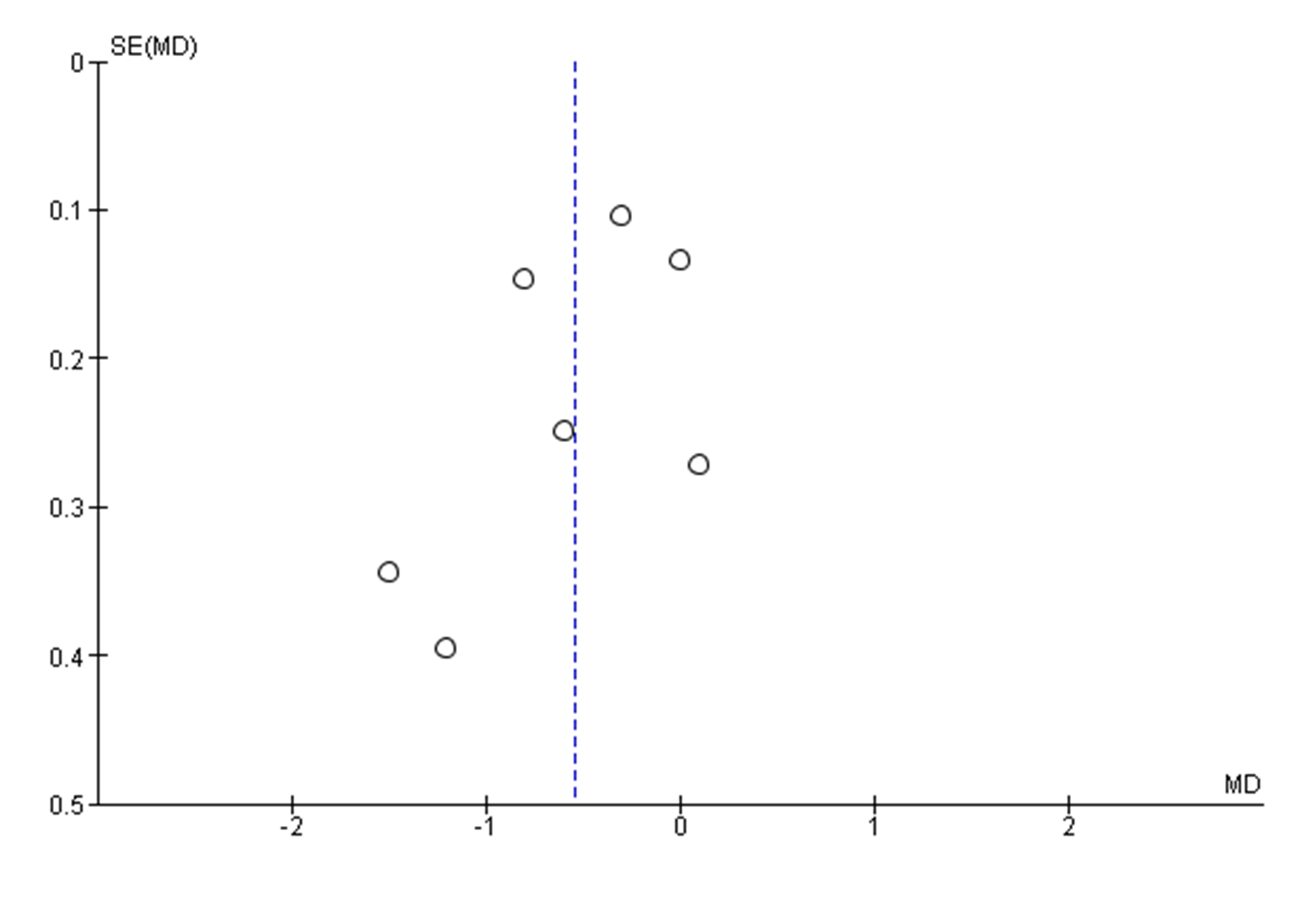

Supplement: S1 Fig — MD, mean difference. (TIF) [file pone.0266917.s001.tif]
