# Supplementary material for: Japanese traditional Kampo medicine bofutsushosan improves body mass index in participants with obesity: A systematic review and meta-analysis
Source: PLoS One. 2022 Apr 13;17(4):e0266917. doi: 10.1371/journal.pone.0266917 (PMC9007387; doi:10.1371/journal.pone.0266917)
Supplement: S1 Table — (DOCX) [file pone.0266917.s002.docx]

**S1 Table.**

| Crude drug | Hioki, Namiki, Azushima | Wu | Xu | Park |  |
| --- | --- | --- | --- | --- | --- |
| Scutellariae Radix | 2.0 | - | 2.0 | 1.0 |  |
| Glycyrrhizae Radix | 2.0 | 10.0 | 2.0 | 1.0 |  |
| Platycodi Radix | 2.0 | 6.0 | 2.0 | 1.0 |  |
| Gypsum Fibrosum | 2.0 | 15.0 | 2.0 | 1.0 |  |
| Atractylodis Rhizoma | 2.0 | 6.0 | 2.0 | 1.0 |  |
| Rhei Rhizoma | 1.5 | 10.0 | 1.5 | 0.8 |  |
| Schizonepetae Spica | 1.2 | 12.0 | 1.2 | 0.6 |  |
| Gardeniae Fructus | 1.2 | 10.0 | 1.2 | 0.6 |  |
| Paeoniae Radix | 1.2 | 10.0 | 1.2 | 0.6 |  |
| Cnidii Rhizoma | 1.2 | 12.0 | 1.2 | 0.6 |  |
| Angelicae Radix | 1.2 | 12.0 | 1.2 | 0.6 |  |
| Menthae Herba | 1.2 | 12.0 | 1.2 | 0.6 |  |
| Saposhnikoviae Radix | 1.2 | 12.0 | 1.2 | 0.6 |  |
| Ephedrae Herba | 1.2 | 12.0 | 1.2 | 0.6 |  |
| Forsythiae Fructus | 1.2 | 12.0 | 1.2 | 0.6 |  |
| Zingiberis Rhizoma | 0.3 | - | 0.4 | 0.2 |  |
| Aluminum Silicate Hydrate  with Silicon Dioxide | 3.0 | 10.0 | 3.0 | 1.5 |  |
| Natrii Sulfas | 0.7 | 6.0 | 0.8 | 0.4 |  |
| Astragali Radix | - | 8.0 | - | - |  |
| Murase et al; unknown. Values are expressed as content in daily dose (g). | | | | | |
